# Uptake of prostate cancer screening and associated factors among men aged 50 years and above in Lira city, Uganda: a cross-sectional study

**DOI:** 10.1186/s12889-023-15348-w

**Published:** 2023-03-06

**Authors:** Richard Ekwan, Emmanuel Bua, Ritah Nantale, Ronald Opito, Patrick Abingwa, Quraish Sserwanja, Job Kuteesa, David Mukunya

**Affiliations:** 1 School of Public Health, Makerere University, Kampala, Uganda; 2 Department of Surgery, Mbale regional referral hospital, Mbale, Uganda; 3 Department of Nursing, Busitema University, Mbale, Uganda; 4 Department of Public Health, Soroti University, Soroti, Uganda; 5 Department of Surgery, Busitema University, Mbale, Uganda; 6 Department of Programmes, GOAL, Khartoum, Sudan; 7 Department of Surgery, Mulago National Referral Hospital, Kampala, Uganda; 8 Department of Community and Public Health, Busitema University, Mbale, Uganda; 9 Department of Research, Nikao Medical Center, Kampala, Uganda

**Keywords:** Prostate cancer, Men, Screening, Uganda

## Abstract

**Background:**

Prostate cancer is the most common cancer among men globally, with over 1.2 million cases reported in 2018. About 90% of men with prostate cancer are diagnosed when the disease is in an advanced stage. We assessed the factors associated with the uptake of prostate cancer screening among men aged ≥ 50 years in Lira city.

**Methods:**

This was a cross-sectional study involving 400 men aged ≥ 50 years in Lira city who were sampled using multistage cluster sampling method. Uptake of prostate cancer screening was defined as the proportion of men who received prostate cancer screening in the past one year prior to the interview. Multivariable logistic regression analyses were performed to assess the factors associated with the uptake of prostate cancer screening. Data were analyzed using Stata version 14.0 statistical software.

**Results:**

Of the 400 participants, only 18.5% (74/400) had ever been screened for prostate cancer. However, 70.7% (283/400) were willing to screen/rescreen if provided with the opportunity. Majority of the study participants, 70.5% (282/400) had ever heard about prostate cancer, mostly from a health worker (40.8% (115/282)). Less than half of the participants had high knowledge of prostate cancer. The factors that were significantly associated with prostate cancer screening were age ≥ 70 years, Adjusted Odds Ratio (AOR) 3.29: 95% Confidence Interval (CI): 1.20-9.00) and having a family history of prostate cancer, AOR 2.48 (95%CI: 1.32–4.65).

**Conclusion:**

There was low uptake of prostate cancer screening among men in Lira City, but majority of men were willing to screen. We encourage policymakers in Uganda to ensure prostate cancer screening services are readily available and accessible by men so as to improve on early identification and treatment of the disease.

## Introduction

Prostate cancer, defined as adenocarcinoma of the prostate gland, is the most common cancer among men globally, with over 1.2 million cases reported in 2018 [1]. Africa has a prostate cancer incidence rate of 26.6 per 100,000 and in sub-Saharan Africa, about 70,000 cases of prostate cancer are reported annually [1, 2]. In Uganda, prostate cancer is also the most common cancer among men with an age-standardized incidence rate of 41.6 per 100,000 [3].

The high morbidity due to cancer in Uganda is attributed to the late presentation of the disease [4]. Most prostate cancer cases referred to Uganda Cancer Institute (UCI) for treatment are at stage IV [5]. Late presentation reflects the lack of access to early diagnosis and treatment, which are signs of the poor status of the cancer care system in the country [4, 5]. Prostate cancer screening could assist in detecting cancer at an early stage when it can easily be cured [6]. Cervical cancer screening in developed countries has shown that primary screening generally detects more than 90% of all cancer cases before they metastasize to other regions of the body system [7], and this same approach can be applied to prostate cancer as recommended by WHO general approach to prioritize and invest in early diagnosis of cancers [8]. Studies have revealed predictors for prostate cancer screening as ever heard about prostate cancer, family history of prostate cancer, higher socioeconomic status, employment status, use of complementary medicine, belief in screening efficacy, awareness of anyone who has undergone prostate cancer screening, and having regular visits to a doctor [9–12]. Early diagnosis is the best alternative for the many cancers that cannot yet be prevented and those that occur despite prevention[13]. However, currently, in most low-and middle-income countries, cancer is diagnosed at an advanced stage, when treatment is generally less effective, more expensive, and more disabling [8].

In Uganda, a study by Nakandi et al. in 2013 revealed that only 22.9% of Ugandan men considered getting a serum PSA test and only 3.5% had ever undergone a serum PSA test for prostate cancer screening [14]. In Lira city, there is paucity of data on Prostate cancer screening. Early screening could be helpful for proper planning of treatment plans since Lira city and other rural areas have no/few cancer treatment centers and need to travel long distances for cancer treatment. Therefore, this study aimed at identifying factors associated with the uptake of prostate cancer screening among men aged ≥ 50 years in Lira city in Uganda.

## Materials and methods

### Study design

This was a cross-sectional study that used quantitative methods of data collection and analysis.

### Study setting

The study was conducted in Lira City, located in Lango sub-region of Northern Uganda, and formerly a municipality within Lira district (it was elevated to a city council on 7th August 2020). Lira City has a population of 457,805 people. Of these 219,576 (48%) are males while 238,229 (52%) are females. The city has two divisions: Lira City East Division (comprising of the then Adekokwok, Ngetta, and Iwal Sub Counties, Lira Central Division, and Railways division of the then Lira municipality) and Lira City West Division comprising of Ojwina Division, Adyel Division of the then Lira municipality and Lira Sub County. The city has two County Administrative units, 49 Wards, and 235 cell administrative units. West Division has 21 Wards, and 75 villages whereas East Division has 28 Wards, and 161 villages (9).

Lira City has 15 health facilities; 1 Regional Referral Hospital, 2 district hospitals (1 Government and 1 private not-for-profit), 1 Government Health Center (HC) IV, 9 HC IIIs (6 Government and 3 private not-for-profit) and 2 Government HC IIs. Services offered include; out patients’ department, maternal and child health, Pediatrics, Immunization, Surgical services, Diagnostic services, HIV services, Rehabilitative, Palliative, and Education services.

### Study population

Men aged ≥ 50 years and residents of Lira City.

We included men aged ≥ 50 years and had given informed consent. Those who were mentally/physically ill were excluded.

### Sample size determination

Sample size was calculated using Kish and Leslie formula (1965). This study considered a 17.3% proportion of the target population estimated to have prostate cancer by the Uganda Cancer Institute Report, (2014)., with a precision of 5%, 95% confidence intervals and a non-response of 20%. This gave us a sample size of 396 participants.

### Sampling technique

We used multistage cluster sampling technique to reach eventual study participants of 400 men ≥ 50 years in Lira City. Each of the divisions was assigned a sample size of 200 since the population size per division is almost equal. In the 1st stage of sampling, we randomly selected 7 wards (1/3) wards from Lira City West and 9 wards (1/3) from Lira City East to make a total of 15 wards for this study. In the 2nd stage of sampling, at ward level, we randomly sampled 2 Cells from each of the selected wards to make a total of 30 cells included in the study. In the 3rd and final stage of sampling, we worked with the local leaders (LCIs) of the selected cells to list all the eligible males in their area for the interviews. Each village on average had 100 eligible men for the interview, though it varied highly from the city centre having higher numbers compared to the remote wards. We used the sample size proportion to the eligible men’s population per cluster. This number was then reached by using simple random sampling method, where the names of the eligible men listed were written on pieces of paper, mixed thoroughly and the names were chosen randomly from the list and interviewed until the computed number per cell is arrived at.

### Study variables

#### Dependent variables

Uptake of prostate cancer screening was defined as the proportion of men who received prostate cancer screening in the past one year prior to the interview. Men who had prostate cancer screening more than one year ago were not considered as having been screened. Men that had screened for prostate cancer were coded as 1 and those that had not screened were coded as 0.

#### Independent variables. These included

Socio-demographic factors such as age categorized as 50–59, 60–69, 70–79 and ≥ 80years, marital status categorized as married/cohabiting, divorced/separated, never married and widowed, number of sexual partners, religion (Muslim, catholic, protestant, Pentecostal and others).

Socio-economic factors such as employment status reported as employed (both formal and non-formal), education level reported as none, primary, secondary and tertiary levels, and sources of income reported as from salary, business, friends, or children.

Health-seeking behaviors were recorded as the number of hospital visits in the last five years and the reasons for the hospital visits.

Level of nearest health facility from the participant’s home was reported as health center II, III, IV.

Distance from the nearest health facility was reported as < 5 km or more than 5 km.

Alcohol and tobacco use was recorded as using or not.

Familial history of prostate cancer was determined by asking participants whether they had a family member who suffered from prostate cancer.

Knowledge/awareness of prostate cancer risks factors and screening.

For knowledge level scoring, each of the 12 awareness and knowledge questions was scored with either 1 for correct answer or 0 for incorrect/I don’t know the answer, and summed up together. The maximum score was 12 and the minimum was 0. Data were analyzed using Stata version 14.0.

### Participant recruitment and informed consent processes

The local area leaders (LCIs) of the selected cells identified men aged ≥ 50 years in their area and accompanied the research assistants to the homes of the men for the interviews. While at the home of the identified eligible participant, the research assistant ascertained eligibility, created rapport and thoroughly explained to the participant the purpose of the study. Participants who were interested in taking part in the study signed the consent form and voluntarily participated. Interviews were conducted at the participants homestead, in a quiet, comfortable and private location within the homestead. Participants that were not found at home were revisited the following day.

### Data collection

Data were collected by four trained research assistants using a structured questionnaire adapted from literature [15]. The questionnaire contained both open and close-ended questions which were used to gather information on demographic, economic, cultural, institutional, knowledge, and attitude factors related to prostate cancer health-seeking behavior of males ≥ 50 years. The questionnaires contained statements that patients could choose from the options that are applicable or add what is not captured in the questionnaire. The questionnaire was translated into Luo (Lango) for ease of understanding. Each questionnaire was evaluated for completeness by the investigators after the interview session had ended. The questionnaire was also pilot tested to assess its validity before actual data collection.

### Statistical analysis

Data collected were entered into Microsoft excel, then exported to Stata version 14.0 software for cleaning, management, and analysis. Descriptive analysis was performed for sociodemographic characteristics and presented in form of proportions, frequencies, mean and median, while binary logistic regression was performed to determine the strength and directions of association of independent variables with the uptake of prostate cancer screening. Variables with a P < 0.25 at bivariable level, those with biological plausibility and confounders were selected for inclusion into the multivariate model. Multivariable logistic regression was performed to assess the factors associated with the uptake of prostate cancer screening. The significance level was set at 5%. Data were analyzed using Stata version 14.0 statistical software. This analysis involved only results from the closed-ended questions.

## Results

### Sociodemographic characteristics of study participants

Majority of study participants, 50.2% (202/400) were aged 50–59 years, with a median age of 59(IQR: 54–67). Most of them, 67.7% (271/400) were married, 66.5% (266/400) were employed, 40.5% (162/400) attained secondary education and 83.7% (335/400) were residing within 5 km from a health facility. Other characteristics are shown in Table 1.

**Table 1:** Sociodemographic characteristics of study participants

Characteristic	Population, N = 400	Proportions (%)
Age in years		
50–59	202	50.5
60–69	169	42.2
≥ 70	29	7.3
**Religion**		
Catholic	120	30.0
Muslim	97	24.2
Pentecostal	77	19.3
Protestant	106	26.6
**Marital Status**		
Married	271	67.7
Divorced/widow/single	129	32.3
**Number of Sexual Partners**		
None	26	6.5
Monogamous	218	54.5
Polygamous	156	39.0
**Education Level**		
None	44	11.0
Primary	77	19.3
Secondary	162	40.5
Tertiary	117	29.2
**Employment Status**		
Employed	266	66.5
Unemployed	134	33.5
**Source of Income**		
Business/farming	152	38.0
Salary	41	10.3
Free government services	120	30.0
Relatives/friends	87	21.7
**Level of Nearest Health Facility**		
Private Clinic	52	13
Health Centre II	23	5.8
Health Centre III	153	38.2
Health Centre IV	34	8.5
Regional Referral Hospital	138	34.5
**Distance from Nearest Health Facility**		
> 5 km	65	16.3
≤ 5 km	335	83.7
**Alcohol Consumption**		
No	248	62.0
Yes	152	38.0
**Cigarette Smoking**		
No	291	72.7
Yes	109	27.3

### Uptake of prostate cancer screening among men aged 50 years and above in Lira city, Uganda

**Fig. 1:**
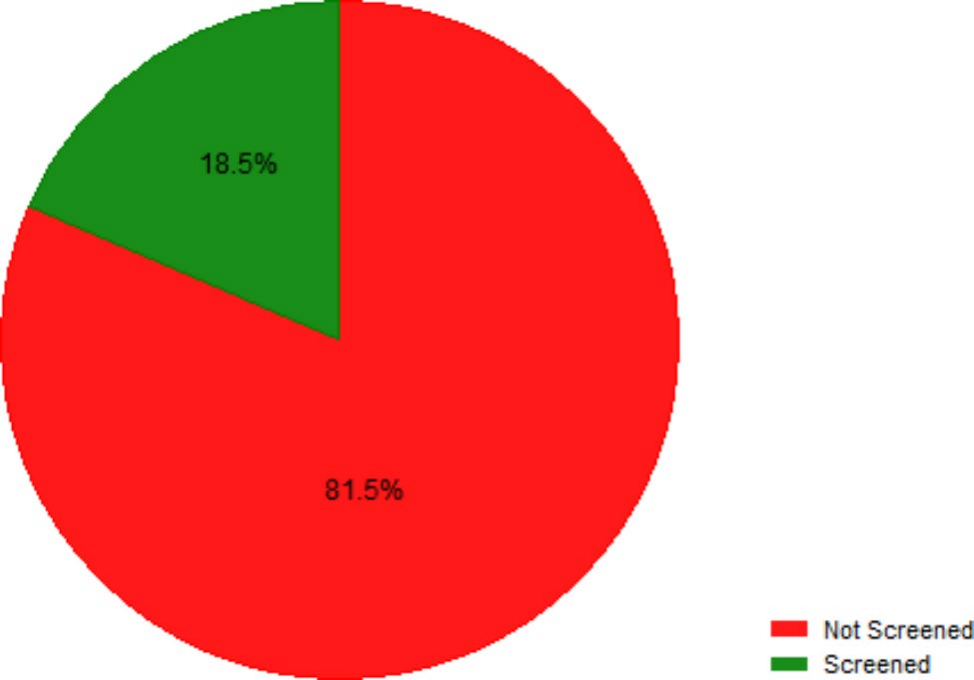
Uptake of prostate cancer screening among men aged ≥ 50 years in Lira city, Uganda

A total of 74 out of 400 participants (18.5%) had ever been screened for prostate cancer (Fig. 1).

### Awareness and knowledge of prostate cancer of men aged 50 years and above in Lira city, Uganda

Majority of the study participants, 70.5% (282/400) had ever heard about prostate cancer (had prior knowledge) and for those who had ever heard about prostate cancer, the most common source of information was from a health worker, 40.8% (115/282). 17.0% (68/400) had a family history of prostate cancer, majority and 70.7% (283/400) were willing to screen/rescreen for prostate cancer.

The median knowledge score was 7 (IQR: 5–9) while the average knowledge score was 6.94 (SD; 2.75). The knowledge level was categorized as low (≤ median) or high (> median) basing on the median score. 45.3% (181/400) of the participants had high knowledge of prostate cancer. (Table 2)

**Table 2:** Level of awareness and knowledge on prostate cancer of men ≥ 50 years in Lira, Uganda

Variable	Population, N = 400	Proportion (%)
**Prior Knowledge of prostate cancer**		
No	118	29.5
Yes	282	70.5
**Source of Information (N = 282)**		
Health worker	115	40.8
Friend/family	77	27.3
Radio/Newspapers	60	21.3
Others	30	10.6
**Family history of prostate cancer**		
No	332	83.0
Yes	68	17.0
**Willingness to screen**		
No	117	29.3
Yes	283	70.7
**Knowledge Level**		
Low	219	54.7
High	181	45.3

### Factors associated with prostate cancer screening among men aged 50 and above years in Lira city, Uganda

The factors that were significantly associated with prostate cancer screening were; age ≥ 70years, Adjusted Odds Ratio (AOR) 3.29 (95% Confidence Interval (CI): 1.20-9.00, *P*-value = 0.020), and having a family history of prostate cancer, AOR 2.48(95%CI: 1.32–4.65, *P*-value = 0.005). (Table 3)

**Table 3:** Factors associated with prostate cancer screening among men aged ≥ 50 years in Lira city, Uganda

Characteristic	Population, n (%), N = 400	Prostate Cancer Screening		COR (95% CI)	AOR (95%CI)	*P*-Value
		Screened, N = 74, n(%)	Not screened, N = 326, n(%)			
**Age in years**						
50–59	202(50.5)	42(20.8)	160(79.2)	1.00	1.00	
60–69	169(42.2)	22(13.0)	147(87.0)	0.57(0.32-1.00)	0.68(0.37–1.26)	0.222
≥ 70	29(7.3)	10(34.5)	19(65.5)	2.01(0.87–4.63)	**3.29 (1.20-9.00)**	**0.020**
**Religion**						
Catholic	120(30.0)	26(21.7)	94(78.3)	1.00	1.00	
Muslim	97(24.2)	12(12.4)	85(87.6)	0.51(0.24–1.07)	0.57(0.25–1.29)	0.178
Pentecostal	77(19.3)	11(14.3)	66(85.7)	0.60(0.28–1.30)	0.80(0.35–1.84)	0.599
Protestant	106(26.6)	25(23.6)	81(76.4)	1.12(0.60–2.08)	1.52(0.76–3.04)	0.235
**Education Level**						
None	44(11.0)	5(11.4)	39(88.6)	1.00	1.00	
Primary	77(19.3)	14(18.2)	63(81.8)	1.73(0.58–5.19)	1.30(0.38–4.41)	0.672
Secondary	162(40.5)	30(18.5)	132(81.5)	1.77(0.64–4.88)	1.69(0.51–5.60)	0.393
Tertiary	117(29.2)	25(21.4)	92(78.6)	2.12(0.76–5.94)	1.66(0.45–6.07)	0.446
**Employment Status**						
Employed	266(66.5)	54(20.3)	212(79.7)	1.00	1.00	
Unemployed	134(33.5)	20(14.9)	114(85.1)	0.69(0.39–1.21)	0.81(0.38–1.70)	0.572
**Source of Income**						
Business/farming	152(38.0)	27(17.8)	125(82.2)	1.00	1.00	
Salary	41(10.3)	12(29.3)	29(70.7)	1.92(0.87–4.23)	1.63(0.64–4.20)	0.308
Free government services	120(30.0)	17(14.2)	103(85.8)	0.76(0.39–1.48)	0.65(0.29–1.46)	0.296
Relatives/friends	87(21.7)	18(20.7)	69(79.3)	1.21(0.62–2.35)	1.76(0.84–3.68)	0.136
**Level of nearest health facility**						
Private Clinic	52(13.0)	10(19.2)	42(80.8)	1.00	1.00	
Health Centre II	23(5.8)	5(21.7)	18(81.7)	1.17(0.35–3.90)	1.31(0.35–4.84)	0.689
Health Centre III	153(38.2)	28(18.3)	125(81.7)	0.94(0.42–2.10)	0.90(0.38–2.14)	0.811
Health Centre IV	34(8.5)	13(38.2)	21(61.8)	2.60(0.98–6.91)	2.85(0.97–8.39)	0.058
Regional Referral Hospital	138(34.5)	18(13.0)	120(87.0)	0.63(0.27–1.47)	0.70(0.28–1.75)	0.439
**Cigarette Smoking**						
No	291(72.7)	47(16.2)	244(83.8)	1.00	1.00	
Yes	109(27.3)	27(24.8)	82(75.2)	1.71(1.00-2.92)	1.75(0.97–3.16)	0.064
**Family history of prostate cancer**						
No	332(83.0)	52(15.7)	280(84.3)	1.00	1.00	
Yes	68(17.0)	22(32.4)	46(67.6)	2.58(1.43–4.64)	**2.48(1.32–4.65)**	**0.005**

*Bold *= Significant with P-value < 0.05, COR = Crude Odds ratio, AOR = Adjusted Odds ratio. All factors are adjusted for each other*

## Discussion

This study assessed the level of uptake of prostate cancer screening and the factors associated with its uptake in an urban setting. We found out that the level of prostate cancer screening among men in Lira City was very low (18.5%). This finding is worrying and demonstrates that more efforts need to be put in place to ensure that prostate cancer screening is readily available, accessible, and acceptable by the men at risk. Moreover, we conducted the study among slightly older men 50 years and above residing in a city, who should have received at least one screening in the last year. This finding of low uptake is not unique to Uganda only, in fact, an earlier study in a tertiary hospital in Nigeria found less than 10% of study participants had received any form of prostate cancer screening [16], indicating a low level of uptake. Similar observations were made in Dare Salam, Tanzania where only 7.7% of the study participants had ever been screened for prostate cancer [17].

A recent study in a rural community in Kenya found an extremely low uptake where only 5% of study participants had ever received prostate cancer screening despite the fact that majority have ever heard about prostate cancer [15]. The extremely low uptake of the service could have been related to the accessibility of the service, being in a rural setup. Studies in other sub-Saharan African countries have equally demonstrated low uptake of prostate cancer screening ranging from 10% in Southwest Nigeria [18] to 13% in Zambia [19]. The highest reported level of prostate cancer screening in SSA in the general population was in a highly developed Lagos city of Nigeria at 21% [20]. One single study in sub–Saharan Africa which reported high prostate screening at 27% was among health workers in Kenya [21]. This makes it worrying if even health workers who are presumed to be knowledgeable about prostate cancer risks and the need for early screening and detection are not able to take up the screening. This further stress the need to have screening services for men at risk readily available and accessible. Studies in developed countries have equally shown low levels of prostate cancer screening among randomly selected men in the general population, with less than a third (only 30%) of study participants in Italy having ever been screened [22].

Our study found that majority (70.5%) of participants had ever heard about prostate cancer and 70.7% were also willing to screen or rescreen if provided with the opportunity. This is a promising finding as it presents an opportunity for the government to scale up prostate cancer screening and early detection in the community. The study also found that the most common source of information is from a health worker, which implies significant efforts which have been put in by the government to improve knowledge and awareness of prostate cancer in the community. These findings show a significant improvement in the knowledge of prostate cancer among men dwelling in Ugandan cities as an earlier study done in Kampala found poor knowledge and misconceptions about prostate cancer and screening in the city, with the most common source of information by then being mass media [14]. Poor knowledge and awareness of prostate cancer have equally been reported in earlier studies conducted in sub-Saharan African countries such as one in Ouagadougou, Burkina Faso where up to two-thirds of study participants did not know about prostate cancer and up to 70% of them did not know about any diagnostic test [23] and Nigeria where less than half of the participants were aware of prostate cancer and only 13.7% were aware of the availability of any screening test [16].

Our finding is similar to a recent study conducted in a rural community in Kenya where they found majority of participants had ever heard about prostate cancer with overall low level of awareness and misconceptions which associated prostate cancer with sexual behaviors [15]. Earlier studies in other sub-Saharan African countries, just like the one done in Kampala have equally indicated very poor knowledge of prostate cancer among men, with less than half of the study participants had ever heard about prostate cancer [17–20, 24]. This demonstrates that in the entire sub-Saharan Africa, there has been a progressive effort in educating the community on prostate cancer and its screening methods. Only one earlier study in a well-developed country (Italy) found that majority of study participants had ever heard about prostate cancer and screening using prostate-specific antigen and most of them had heard about it through their physicians [22].

Our study found the factors that were significantly associated with prostate cancer screening to be age ≥ 70 years and having a family history of prostate cancer. Family history of prostate cancer has been found to be influencing prostate cancer screening in other sub-Saharan African countries as observed in Zambia and Nigeria [19, 25]. This demonstrates the fact that family members of an affected individual affected by prostate cancer also get enough health education regarding the disease, early detection, and its prevention intervention and they are able to follow the health worker’s recommendations. On the other hand, older age was associated with increased awareness, accessibility to testing services, and knowledge of PSA test among Nigerian men [26], an observation similar to our findings and those reported in Dar Es Salam where age > 60years was positively associated with utilization of prostate cancer screening [17]. This could be resulting from the fact that community sensitization on prostate cancer stress that advancing age is a risk factor for prostate cancer and men tend to screen for the disease at an older age.

Other factors previously observed to be affecting the uptake and utilization of prostate cancer in SSA such as education level [17, 18, 20, 21] did not have any association in our study. Socio-economic factors such as owning land and having > 305 USD were observed in neighboring countries of Kenya and Tanzania to be positively associated with prostate cancer screening [15, 17]. However, these were not included in our study, and the only socioeconomic factor assessed was the source of income, which was not significantly associated with the uptake of prostate cancer screening.

## Strengths and limitations

Our study had some limitations as it was a cross-sectional study, therefore the association of dependent and independent variables could not be clearly explained. Additionally, the study was conducted in an urban setting thus our findings may not be representative of men in the rural community. We also faced a challenge of recall bias where very old men aged 85 years and above could fail to recall the recent medical examinations they underwent. Nevertheless, to our knowledge, this is the first study on prostate cancer screening in Lira city, Uganda. The study provides relevant information for designing strategies to improve prostate cancer screening in the community.

## Conclusion

There was low uptake of prostate cancer screening among men in Lira City, but majority of men were willing to screen for prostate cancer. Factors associated with prostate cancer screening were age and family history of prostate cancer. We recommend that further studies should be conducted in this community to establish why there was very low uptake of prostate cancer screening despite the fact that many participants had ever heard about it and got the information from the health workers. Furthermore, there is need to scale up screening services for prostate cancer in the community so as to enable early diagnosis and treatment of the disease and reduce morbidity and mortality from it.

## Data Availability

The datasets used and/or analyzed during the current study are available from the corresponding author on reasonable request.

## List of abbreviations

ACS: American cancer society
AOR: Adjusted Odds Ratio
COR: Crude Odds Ratio
DRE: Digital rectal examination
LRRH: Lira Regional Referral Hospital
PC: Prostate cancer
PSA: Prostate specific antigen
UCI: Uganda cancer institute
